# TGF-β1–induced endothelial-mesenchymal transition: a potential contributor to fibrotic remodeling in atrial fibrillation?

**DOI:** 10.1172/JCI161070

**Published:** 2022-07-01

**Authors:** Arnela Saljic, Eleonora Grandi, Dobromir Dobrev

**Affiliations:** 1 Institute of Pharmacology, West German Heart and Vascular Center, University Duisburg-Essen, Essen, Germany.; 2 Department of Biomedical Sciences, Faculty of Health and Medical Sciences, University of Copenhagen, Copenhagen, Denmark.; 3 Department of Pharmacology, University of California Davis, Davis, California, USA.; 4 Department of Molecular Physiology and Biophysics, Baylor College of Medicine, Houston, Texas, USA.; 5 Montréal Heart Institute and University de Montréal, Medicine and Research Center, Montréal, Quebec, Canada.

## Abstract

Atrial fibrillation (AF) is the most common cardiac arrhythmia worldwide, with an unmet therapeutic need. Fibrotic remodeling, in which collagen-producing atrial fibroblasts play a crucial role, substantially contributes to arrhythmia promotion and progression. In this issue of the *JCI,* Lai, Tsai, and co-authors reveal that TGF-β1 promoted endothelial-mesenchymal transition during AF and put forward the notion that, in the adult heart, atrial fibroblasts can originate from different cellular sources. These important findings extend our understanding of the origin, biology, and function of fibroblasts and offer possibilities for therapeutic targeting of fibrosis in AF.

## Atrial fibrotic remodeling and atrial fibrillation

Atrial fibrillation (AF) is a common arrhythmia that associates with increased morbidity and mortality. Current pharmacological approaches have limited efficacy, with an unmet need for better therapeutics. Moreover, therapies that halt or prevent arrhythmia progression to more persistent and therapy-resistant forms remain unavailable (1). Thus, a better understanding of the underlying mechanisms that maintain AF may lead to more efficient anti-AF therapeutics.

**Figure 1:**
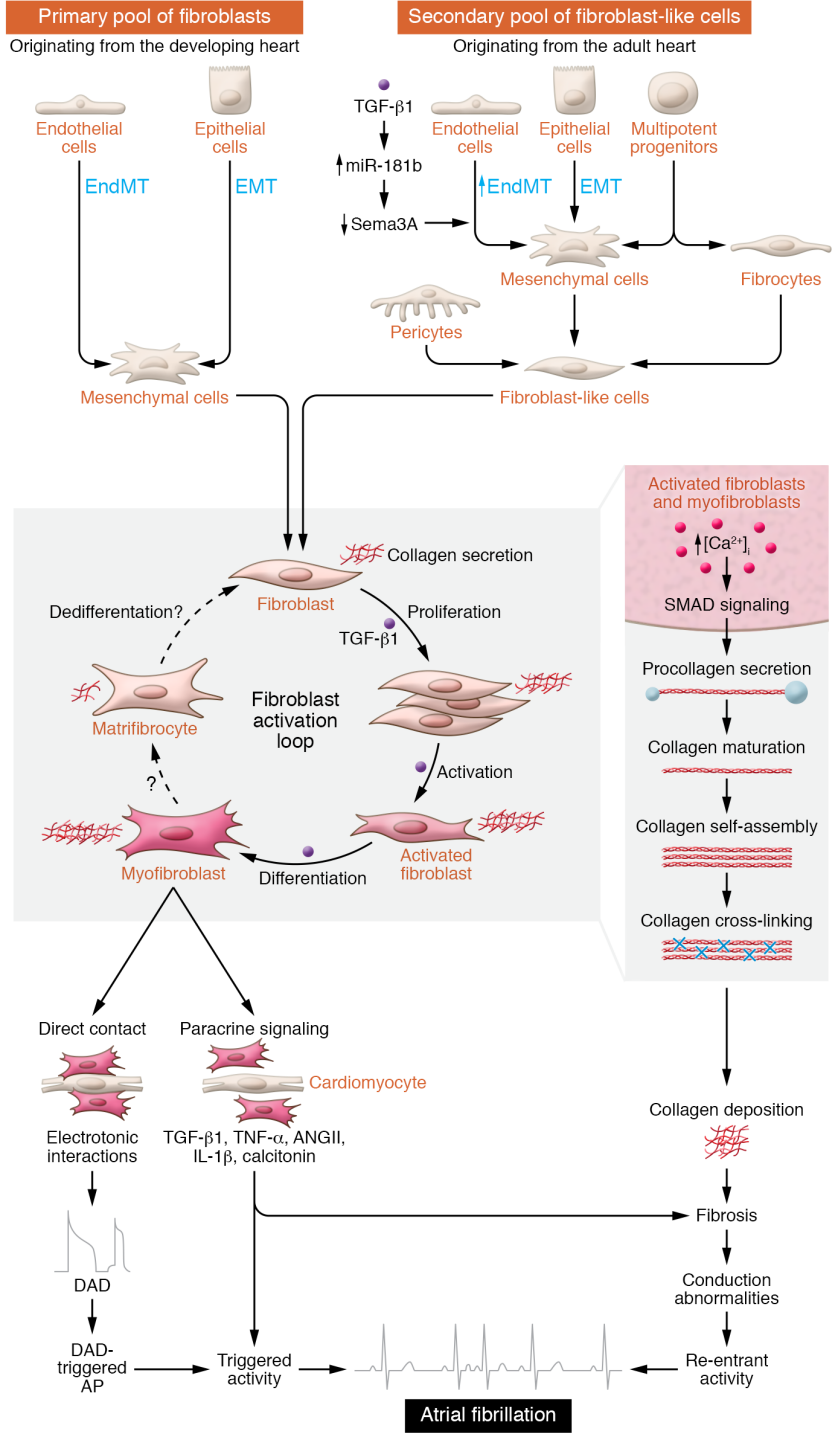
Origin and consequences of fibroblast activation for atrial arrhythmogenesis. Cardiac fibroblasts originate from multiple cellular sources including endothelial and epithelial cells. During development, the primary pool of resident, quiescent cardiac fibroblasts arises from the mesenchyme via EMT and EndMT. Resident fibroblasts are the primary cells responsible for ECM homeostasis. In disease, a secondary pool of fibroblast-like cells arises from various cellular sources including epithelial and endothelial cells via EMT and EndMT. The exact contribution of the secondary pool to fibroblast generation is unknown. Profibrotic stimuli cause the proliferation and differentiation of fibroblasts into myofibroblasts, increasing collagen production. Ca^2+^ influx importantly regulates fibroblast function, and increases in intracellular Ca^2+^ promote the differentiation of fibroblasts into collagen-secreting myofibroblasts. Collagen production steadily increases throughout the fibroblast activation loop and peaks when the fibroblasts have fully differentiated into activated myofibroblasts. Whether the collagen-secreting myofibroblasts differentiate into quiescent matrifibrocytes in the atria is unknown. Pro-collagen is synthesized in the ER and secreted into the extracellular space, where it matures and assembles into collagen fibers. Enhanced collagen deposits result in fibrotic remodeling and conduction abnormalities that promote AF-maintaining reentrant activity. Activated fibroblasts and myofibroblasts secrete signaling molecules and can electrotonically couple with cardiomyocytes to potentially promote delayed afterdepolarization (DAD), DAD-triggered action potentials (AP), and ectopic (triggered) activity that may cause AF.

The fundamental AF mechanisms include ectopic activity, also called triggered activity, which can promote bursts of atrial depolarizations and lead to a pattern known as reentry, in which the abnormal impulse repetitively propagates through a circuit (2, 3). Numerous underlying risk factors and disease conditions promote AF (3, 4) by causing changes in ion channels and electrical properties, autonomic tone, Ca^2+^ handling, and structural remodeling in the atria (2, 3). Cardiac fibrosis is a hallmark of atrial structural remodeling and is characterized by excessive deposition and expansion of extracellular matrix (ECM) components by cardiac fibroblasts and myofibroblasts. Under normal conditions, resident fibroblasts are quiescent and produce less ECM (5) (Figure 1), whereas fibroblast activation and increased deposition of collagen are typical in AF paradigms (2). For instance, atrial fibrosis in patients with AF is approximately four-fold higher in five different atrial regions compared with heart tissue from patients with sinus rhythm (SR), without differences among the regions (6). However, many patients with AF also have valvular heart disease, which per se causes atrial fibrotic remodeling (7). Patients with SR and valvular heart disease have more atrial fibrosis in the left atrium than do patients with coronary artery disease (8). Conversely, hearts from patients with AF and valvular heart disease exhibit more fibrosis in the right atrium compared with hearts from patients with SR (8). Thus, fibrosis appears to be region specific, and further experiments need to determine whether AF is a consequence or a cause of atrial fibrosis. In addition, besides collagen deposition, the dynamic paracrine influences and the electrotonic coupling between fibroblasts and cardiomyocytes could also contribute to the AF-promoting substrate (9, 10) (Figure 1).

## Origin and function of atrial fibroblasts

Cardiac fibroblasts have multiple distinct cellular origins. The primary pool of resident cardiac fibroblasts is of mesenchymal origin arising through epithelial-mesenchymal transition (EMT) and endothelial-mesenchymal transition (EndMT) during embryonic development (Figure 1). In disease, a secondary pool of fibroblast-like cells arises during fibrogenesis and pathological remodeling from different cellular sources, including epithelial and endothelial cells through EMT and EndMT (Figure 1). EndMT was shown to contribute to cardiac fibrosis over a decade ago (11) and has recently been implicated in fibrotic remodeling in human AF (12). In this issue of the *JCI*, Lai, Tsai, and co-authors (13) investigated the mechanisms of EndMT in atrial fibrosis by using human endocardial endothelial cells from human atrial appendages and transgenic mice with cardiac-specific TGF-β1 overexpression. In human endocardial endothelial cells, treatment with TGF-β1 induced EndMT by upregulating miR-181b expression via SMAD2/3 signaling pathways. The higher miR-181b expression caused a reduction of semaphorin 3A (Sema3A), which relieved the inhibition of LIM kinase 1/phosphorylated cofilin (LIMK/p-cofilin) signaling pathways, leading to EndMT (13). While several miRNAs have been implicated in the regulation of atrial fibroblasts (14), Lai, Tsai, and colleagues showed that miR-181b knockdown increased Sema3A expression, thereby reversing fibrosis and reducing AF vulnerability in TGF-β1–overexpressing mice (13). These data suggest that prevention of de novo EndMT by targeting the miR-181b/Sema3A/LIMK/p-cofilin pathway might constitute a potential antifibrotic strategy for AF (13).

The precise role of de novo EndMT for fibroblast generation and fibrosis formation is poorly understood. While some studies support de novo synthesis in adult hearts, others consider EndMT a source of resident cardiac fibroblasts exclusively during embryogenesis (15, 16). This inconsistency could result from the markers used to trace EndMT. Lai, Tsai, and co-authors (13) used common markers of endothelial cells (CD31), fibroblasts, and myofibroblasts (vimentin and α–smooth muscle actin [α-SMA]), along with markers of EndMT (the transcription factors Twist, Snail, and Slug) to demonstrate EndMT. Despite their common use, these markers can nonspecifically label several cell types. Transgenic mouse models are being developed for more accurate lineage tracing, and in vivo cellular tracing in humans using fluorodeocyglucose-PET (FDG-PET) and PET-MRI is emerging (17). Although further validation is needed, the work presented by Lai, Tsai, and colleagues (13) supports the notion that de novo EndMT may occur in the atria, particularly upon TGF-β1 stimulation. Nevertheless, it is unclear how EndMT-derived fibroblast-like cells contribute to fibrogenesis. Do they belong to the collagen-secreting myofibroblast population, or do they exert paracrine effects that indirectly activate resident fibroblasts? Since EndMT was not revealed in previous studies that focused on ventricular fibrosis (11, 15, 16), it is possible that de novo EndMT is an atrium-specific phenomenon. Overexpression of TGF-β1 in mice causes fibrosis in atria only and increases AF susceptibility (18). Atrial fibroblasts are more responsive to TGF-β1 and angiotensin II than are ventricular fibroblasts (19). Thus, atrial-ventricular differences offer a unique opportunity to develop atrium-selective antifibrotic targets.

Besides emerging evidence for a role of EMT and EndMT reactivation in profibrotic remodeling, activation of resident fibroblasts under disease conditions and during AF is a common finding. Given the paracrine or autocrine effects of profibrotic molecules, cardiac fibroblasts can proliferate and differentiate into collagen-secreting myofibroblasts (Figure 1). Pro-collagen is synthesized in myofibroblasts and secreted as soluble pro-collagens into the extracellular space, where it is processed, assembled into fibrils, and cross-linked (Figure 1). Several cross-linking enzymes including lysyl oxidases are upregulated in the atria of patients with AF (20). Cross-linking of elastin is irreversible, whereas cross-linking of collagen appears to be reversible, constituting a viable antifibrotic target.

Whether changes in collagen composition play a causal role in AF pathophysiology warrants direct inquiry. The heart contains mainly type I (approximately 85%) and type III (approximately 15%) collagen. Collagen type I forms thicker and stiffer fibers, while collagen type III forms finer reticular fibers that are more compliant, increasing tissue elasticity (21). Changes in the type I to type III collagen ratio have been found to occur with cardiac dysfunction in human and experimental studies (22), and upregulation of nonfibrillar collagen type VI was reported in patients with AF (23). Although nonfibrillar collagens are not organized in larger fibrillar bundles, they can still interact with type I and type III collagens. TGF-β1 and angiotensin II increase the synthesis and secretion of fibrillar and nonfibrillar collagens, and collagen type IV promotes the differentiation of fibroblasts into myofibroblasts (23). Thus, additional collagen types may exist in the heart and could contribute to fibroblast dysfunction. Future work should directly address this hypothesis.

## Therapeutic considerations

Therapeutic targeting of cardiac fibrosis is challenging. Besides possible targeting of de novo fibroblast synthesis, as elegantly shown in the work by Lai, Tsai, and colleagues (13), the unique electrical properties of fibroblasts open up several therapeutic possibilities. Experimental data point toward a central role of Ca^2+^ entry through store-operated Ca^2+^ channels (SOCs) and transient receptor potential (TRP) channels in the activation and proliferation of fibroblasts and their differentiation into myofibroblasts (24) (Figure 1). Thus, targeting fibroblast Ca^2+^ handling might be another therapeutic option. There is evidence for a direct electrotonic coupling between myofibroblasts and cardiomyocytes that causes cardiomyocyte depolarization, potentially promoting triggered activity (10, 25). Finally, cardiac myofibroblasts are able to differentiate into matrifibrocytes, a phenotype recently defined in mouse and human scar tissue that is characterized by a loss of proliferation ability, a decrease in α-SMA expression, and a reduced capacity to secrete collagen (25). Hence, future work should assess whether matrifibrocytes exist in atrial tissue and whether and how they influence the susceptibility to fibrotic remodeling and AF.

Lai, Tsai, and co-authors (13) add important insights into the complex origin and function of cardiac fibroblasts and offer possibilities for therapeutic targeting of fibrosis in AF.

## Version 1 07/01/2022

Electronic publication
